# A rare case of penile granulomatosis with polyangiitis: case report and multidisciplinary management approach

**DOI:** 10.3389/fruro.2026.1790745

**Published:** 2026-03-18

**Authors:** Matteo Coschignano, Nicolò Schifano, Benedetta Pennella, Sara Baldini, Ilaria Zais, Alessio Villano, Paolo Capogrosso, Gabriele Antonini, Alberto Batticciotto, Antonella Cappelli, Federico Dehò

**Affiliations:** 1 Urology Department, Azienda Socio-Sanitaria Territoriale (ASST) Sette Laghi – Circolo and Fondazione Macchi Hospital, Varese, Italy; 2 Department of Medicine and Surgery, University of Insubria, Varese, Italy; 3 Rheumatology Unit, Internal Medicine Department, Azienda Socio-Sanitaria Territoriale (ASST) Sette Laghi – Circolo and Fondazione Macchi Hospital, Varese, Italy

**Keywords:** genitourinary manifestations, granulomatosis with polyangiitis with penile involvement, hyperbaric oxygen therapy, methicillin-resistant *Staphylococcus aureus*, necrotizing vasculitis, skin grafting

## Abstract

Granulomatosis with polyangiitis (GPA) is a rare systemic vasculitis presenting with penile involvement in approximately 1% of male patients diagnosed with the disease, making genital manifestations exceedingly rare in this context. We aimed to describe here the case of a 34-year-old male patient with a history of GPA presenting to our attention for a genital ulcer that required a coordinated multidisciplinary management. A 34-year-old man with a known history of systemic GPA presented to the Accident and Emergency (A&E) Department with a dorsal penile shaft abscess that progressed into a necrotic ulcer consistent with a localization of GPA (active vasculitis) based on histopathological examination. The patient underwent escharotomy and split-thickness skin grafting (STSG), which failed to take possibly due to local infection with methicillin-resistant *Staphylococcus aureus* (MRSA) and/or suspected persistent vasculitic activity. Targeted antimicrobial therapy, corticosteroid-based immunosuppression, and hyperbaric oxygen therapy (HBOT) were subsequently implemented, eventually obtaining secondary-intention wound healing. The total hospital stay was 21 weeks. The patient reported a penile curvature of approximately 110° during erection, resulting in the inability to engage in penetrative intercourse. The Visual Analogue Scale (VAS) for the aesthetic and functional outcomes was reported as unsatisfactory (i.e., 2 out of 5). Penile involvement in GPA necessitates a coordinated multidisciplinary approach. Surgical management of GPA-associated large penile skin defects using STSG should be attempted to minimize the hospital stay and to optimize the aesthetic and functional outcomes; however, patients should be informed about a higher likelihood of complications in this setting. The second-intention healing process for large genital skin defects due to GPA may require long hospital stays and may be associated with severe functional issues despite optimal medical management. HBOT may be beneficial in select cases of GPA with genital involvement. Further studies are needed to develop evidence-based guidelines for the management of GPA genital lesions.

## Introduction

Granulomatosis with polyangiitis (GPA) is a rare small- to medium-sized vessel systemic vasculitis characterized by a necrotizing granulomatous inflammation (1) that can involve the respiratory tract, the skin, the eyes, the kidney, and, rarely, the external genitalia (2). A prevalence of three cases per 100,000 (3) people has been reported for GPA (2), affecting both men and women equally, and with a mean age of onset of 41 years (4).

The disease is typically associated with the identification in the serum of both the autoantibody anti-neutrophil cytoplasmic antibody (c-ANCA) and the antibodies against proteinase 3 (PR3), a serine proteinase found in neutrophils (2). The diagnosis of GPA is mainly based on the identification of a combination of clinical and histopathological features, including necrotizing granulomas of the upper and lower respiratory tracts, systemic necrotizing vasculitis of the small vessels, and renal involvement with rapidly progressing necrotizing glomerulonephritis leading to renal failure (4).

The etiology of GPA remains unknown (4). Although a number of familial cases involving first-degree relatives have been reported (5), such instances are anecdotic (2, 4). The frequent involvement of both the upper and lower airways in affected individuals suggests that the disease may result from a complex interplay of some possible inhaled antigens in an individual with a genetic susceptibility to an altered immune reactivity, as well as some other possible host-related factors (6). The inhalation of the triggering agent(s) is hypothesized to lead to extensive neutrophil activation, inducing the translocation of the PR3 to the cell membrane and elevating the levels of the c-ANCA antibodies, which form circulating immune complexes (2).

With regard to genetic predisposition, a notable association has been observed with the *HLADPB1* allele (6).

It has been recognized that some forms of the disease may initially present as localized even for prolonged periods before multi-organ involvement occurs (6). Such limited presentations may involve, e.g., the respiratory tract, but not the kidneys, or *vice versa*. A limited superficial form of GPA has been described as well, where the necrotizing vasculitis is limited to the skin and mucosa before systemic involvement takes place (5). Male urogenital involvement has been anecdotally reported in the literature, with some 20 cases of patients with GPA presenting with genital skin involvement having been reported (7, 8), with an estimated prevalence of 1% among the male patients with a GPA diagnosis (9). Painless genital skin ulceration was the presenting symptom in almost all of the cases, either at disease onset and during flares (10, 11). Genital ulcers are usually recurrent and, in some cases, accompanied by local edema and/or regional lymphadenopathy (7, 8, 10–12).

We aimed to discuss here a case of GPA presenting with penile skin involvement that required a multidisciplinary approach, including surgical management, medical therapy, and hyperbaric oxygen therapy (HBOT).

## Case description

A 34-year-old male patient presented to the Accident and Emergency (A&E) Department in May 2025 complaining of dysuria and pyuria accompanied by edema of the urethral meatus and penile shaft. He was a heavy smoker and continued to smoke even during the whole hospital stay and after hospital discharge. He was already known for a history of systemic GPA.

**Figure 1:**
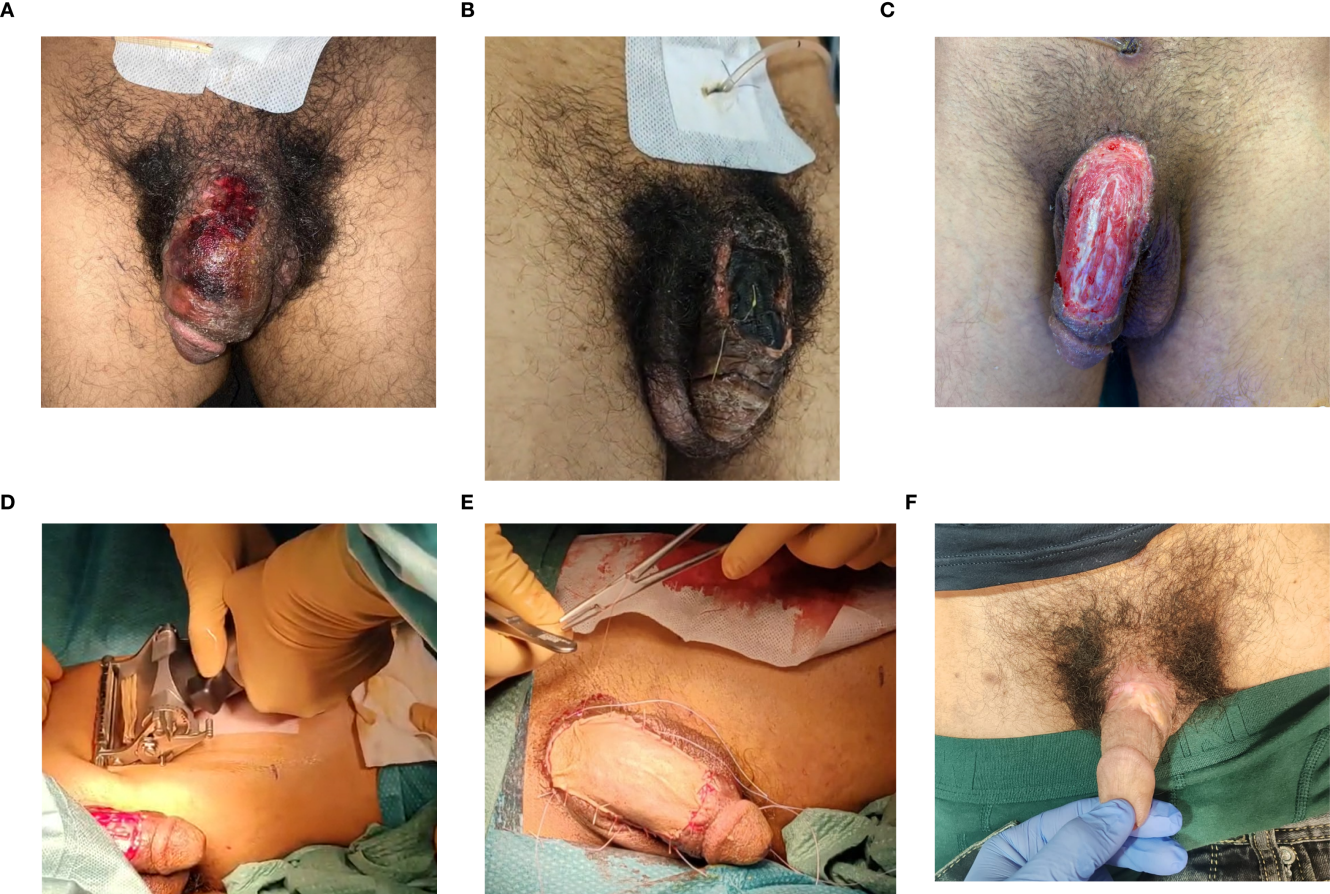
Evolution of the penile lesion surgical management. **(A, B)** Photographs showing the evolution of the necrotic eschar during the hospital stay. **(C–E)** The penile skin defect and its coverage using the skin graft. **(F)** The final result after the second-intention healing process.

At disease onset, the patient presented with severe headache and constitutional symptoms: fatigue, loss of appetite, recurring fevers, and inflammatory arthralgia that evolved during follow-up into migrant arthritis. Baseline magnetic resonance imaging (MRI) revealed a large lesion, 36 mm × 50 mm, with intense contrast enhancement extending into the retropharyngeal space and additional masses with pathological enhancement at the level of the pterygoid fossa, maxillary sinus, and orbital structures. The biopsy mapping procedure performed under general anesthesia revealed histopathological findings consistent with the features of GPA. Blood tests revealed elevated inflammatory markers, while myeloperoxidase (MPO) and the PR3 and ANCA autoantibodies were negative. Further diagnostic investigations excluded pulmonary, renal, cardiac, and nervous system involvement. The baseline Birmingham Vasculitis Activity Score (BVAS) was 9 and the Vasculitis Damage Index (VDI) was 3. The patient had received intravenous rituximab since 2019, with the last rituximab infusion and methotrexate dose administered 1 year before presentation to the A&E Department due to loss to follow-up. Empirical therapy with fluoroquinolone and doxycycline was initiated, and the patient was discharged. He subsequently developed acute urinary retention (AUR) requiring suprapubic catheter (SPC). Computed tomography (CT) of the abdomen and pelvis revealed significant edematous swelling of the penis and surrounding subcutaneous tissues, while the corpora cavernosa appeared structurally preserved. An 8-mm dorsal penile shaft skin suppurative abscess developed, along with the appearance of multiple reactive inguinal lymph nodes that were predominantly represented on the right groin. Laboratory evaluation revealed leukocytosis with markedly elevated inflammatory markers: C-reactive protein (CRP) of 87 mg/dl and erythrocyte sedimentation rate (ESR) of 120 mm/h. The blood, urine, and urethral swab cultures were negative. Serological testing excluded *Toxoplasma gondii*, syphilis, human immunodeficiency virus (HIV), varicella-zoster virus, parvovirus B19, hepatitis B and C, cytomegalovirus, and herpes simplex virus. QuantiFERON testing was negative. Owing to inadequate response to antibiotics, Internal Medicine was consulted, followed by a rheumatology assessment. The abscess rapidly evolved into an ulcerative lesion, which was covered with a black necrotic eschar (**Figures 1A, B**). A bedside penile skin biopsy under local anesthesia (LA) was performed to investigate vasculitis. Histopathology revealed a cutaneous ulcer with underlying necrotizing vasculitis, predominantly lymphocytic, with epithelioid granulomas and occasional multinucleated giant cells. Following rheumatology recommendations, the patient received intravenous methylprednisolone at 1 mg/kg, transitioning to oral prednisone at 50 mg/day. An escharotomy of the necrotic tissues was therefore performed under LA in the surgical theater, leaving a dorsal shaft penile skin defect of approximately 3 cm × 6 cm from the base of the penis to the distal shaft, which was initially dressed with paraffin-soaked bandages. The skin defect was curetted bedside under LA to remove necrotic tissues and exudates using a no. 10 blade every 48 h for a week, until a cleansed and vascular wound bed was obtained (**Figure 1C**). A 0.3-mm split-thickness skin graft (STSG) of adequate dimensions to cover the defect (**Figure 1D**) was harvested from the left thigh and applied on the defect, and the graft was covered with tie-over dressing for graft application (TODGA), which was left in place for 1 week after hospital discharge (**Figure 1E**). At re-evaluation, 1 week after surgery, the TODGA dressing appeared wet and the wound was oozing purulent discharge; therefore, it was removed, revealing graft failure. The patient was therefore readmitted and commenced on an empirical antibiotic treatment and wound dressing every 48 h. The wound swab cultures initially isolated methicillin-resistant *Staphylococcus aureus* (MRSA), prompting initiation of targeted antimicrobial therapy. HBOT was therefore commenced, consisting of 16 weekly sessions. A corticosteroid-based immunosuppressive therapy was commenced with intravenous methylprednisolone at 1 mg/kg, based on the histopathology results of concomitant active vasculitis. Discontinuation of the antibiotic treatment was allowed when the repeated wound cultures were negative. The SPC was removed after a successful trial-without-catheter (TWOC) attempt, when the wound improvement allowed for easier management of the dressings. The patient was discharged with instructions for daily wound care and scheduled follow-up to monitor healing by secondary intention. Complete penile skin re-epithelialization took 25 weeks, including 21 weeks inpatient, achieving a satisfactory aesthetic result from a surgical perspective (**Figure 1F**). The patient subsequently continued therapy with rituximab and initiated leflunomide treatment, aiming to achieve a steroid-sparing effect. The patient was treated with methotrexate 15–20 mg once weekly as a steroid-sparing agent and for the control of joint manifestations of the disease: inflammatory arthralgia and migratory arthritis since diagnosis. When gastrointestinal intolerance occurred, leading to self-discontinuation of methotrexate, we decided to switch to leflunomide in consideration of the joint involvement. Although leflunomide is not commonly used as a disease-modifying anti-rheumatic drug (DMARD) in GPA, a number of studies in the literature have reported its efficacy (13, 14). Regarding functional outcomes, the patient reported a curvature of approximately 110° during erection, which results in an inability to engage in penetrative intercourse (images not provided). Consequently, the use of a vacuum pump was recommended as part of the management plan. The VAS for the aesthetic and functional outcomes was subjectively reported as unsatisfactory by the patient himself (VAS = 2 out of 5). Written informed consent for inclusion of the anonymized data and images in this study was obtained.

## Diagnostic assessment

On presentation to the A&E Department, the patient exhibited pronounced dorsal ulcerations of the penis, accompanied by diffuse edema and localized hematoma. Careful inspection revealed no overt signs of corporal involvement, which was subsequently confirmed by contrast-enhanced CT. Imaging demonstrated significant penile edema with the corpora cavernosa structurally intact, providing crucial information to guide further management. The blood and urine cultures, urethral swabs, and serology for sexually transmitted infections were all negative, making an infectious cause of the ulcers unlikely. Histopathological analysis of a biopsy specimen revealed necrotizing vasculitis, confirming a diagnosis consistent with GPA. This finding highlights the rare manifestation of systemic vasculitis in the genital region, an unusual and clinically challenging presentation. The diagnostic journey was complex, reflecting both the rarity of penile involvement in GPA and the need to consider a broad differential. Differentiating sexually transmitted infections, penile malignancies, and vasculitic disorders posed a diagnostic challenge. Syphilis and genital herpes were excluded via negative serology and lesion timing. Chancroid and lymphogranuloma venereum were ruled out due to the absence of recent sexual exposure and histopathology, while donovanosis was unlikely without progressive nodules or bleeding ulcers. Behçet’s disease was considered, but was excluded due to the lack of systemic manifestations and histological confirmation. The clinical course, age, and history argued against neoplasia. Limited access to specialized testing and cultural factors delayed the diagnosis. Integration of the clinical, radiological, and histopathological findings confirmed active penile GPA. This case highlights the need to consider systemic vasculitis at atypical sites, as delayed recognition may cause tissue damage, functional impairment, and long-term complications. Early diagnosis and targeted therapy are essential to optimize outcomes.

## Discussion

To the very best of our knowledge, this report represents the first case in which a GPA genital ulcer was managed using a multidisciplinary approach, including skin grafting coverage of the defect and HBOT. GPA is a rare systemic necrotizing vasculitis predominantly affecting small- to medium-sized vessels, commonly involving the respiratory tract and kidneys. Cutaneous and genitourinary involvement in GPA is rare, but can create significant diagnostic and therapeutic challenges. This case illustrates severe penile skin involvement, highlighting the importance of multidisciplinary management and tailored therapy. Penile GPA may mimic infections or malignancies, delaying diagnosis. The observed skin graft failure likely reflected ongoing small-vessel vasculitis, interfering with the graft healing stages such as inosculation or revascularization. HBOT, although not a standard treatment for GPA, was implemented in this case in an effort to enhance the tissue oxygenation and support the wound healing process, showing in this case a favorable effect, although no control group was available here for a comparison *vs*. no intervention. The optimization of the corticosteroid therapy was critical to control the inflammatory process and possibly to enhance the healing process. A multidisciplinary management approach integrating medical and surgical expertise should always be implemented for the treatment of any GPA-associated genital involvement. Surgical management of large penile skin defects using skin grafts should be attempted to minimize the hospital stay and to optimize the aesthetic and functional outcomes; however, the patient should always be thoroughly informed about a higher likelihood of complications, such as possible graft infection or graft failure. Patients should always be informed that the second-intention healing process for large genital skin defects due to GPA may require long periods of time and long hospital stays and may be associated with severe functional issues despite optimal medical management. HBOT is feasible in select cases of GPA genital involvement and appeared to provide favorable effects on the healing process of the penile skin defect in this case. Further studies are needed to better characterize the genital skin manifestations of GPA and to develop evidence-based guidelines for their management.

## Patient perspective

The patient was fully informed of the complexity of his condition and the potential complications associated with both medical and surgical interventions. He agreed to be managed by a multidisciplinary uro-rheumatology team, with the dual aims of controlling his underlying rheumatologic disease and promoting healing of the dorsal penile lesion. Despite optimal planning, poor therapy adherence, post-grafting skin infections, and procedure-related complications resulted in suboptimal functional outcomes. Scar formation led to a penile curvature of approximately 110° during erection, significantly impairing sexual function. Although the underlying rheumatologic disease is currently well controlled, the patient continues to experience persistent sexual dysfunction. To address this, the use of a vacuum erection device was recommended as an adjunctive measure to improve functional outcomes. The patient subjectively rated the aesthetic and functional results as unsatisfactory, reporting a VAS score of 2 out of 5.

## Data Availability

The original contributions presented in the study are included in the article/supplementary material. Further inquiries can be directed to the corresponding author.
